# The Role of Depletion of Gut Microbiota in Osteoporosis and Osteoarthritis: A Narrative Review

**DOI:** 10.3389/fendo.2022.847401

**Published:** 2022-03-28

**Authors:** Zhiyuan Guan, Liying Luo, Shengfu Liu, Zhiqiang Guan, Qinggang Zhang, Xu Li, Kun Tao

**Affiliations:** ^1^ Department of Orthopedics, The Shanghai Tenth People’s Hospital of Tongji University, Shanghai, China; ^2^ Tongren Hospital, Shanghai Jiao Tong University School of Medicine, Shanghai, China; ^3^ Department of Dermatology, Xuzhou Municipal Hospital Affiliated with Xuzhou Medical University, Xuzhou, China; ^4^ Spine Center, Department of Orthopedics, The First Affiliated Hospital of University of Science and Technology of China, Division of Life Sciences and Medicine, University of Science and Technology of China, Hefei, China

**Keywords:** osteoporosis, osteoarthritis, gut microbiome depletion, bone, immune

## Abstract

Osteoporosis and osteoarthritis are common diseases in an aging society, are considered metabolic diseases, and affect the quality of life of older adults. In addition, the gut microbiome is considered an additional organ to regulate bone metabolism. In the past decade, people have been studying the relationship between gut microbiota and bone metabolism. The role and mechanism of the gut microbiota in regulating bone metabolism is very important to improve the development of osteoporosis and osteoarthritis. Depletion of the gut microbiota as a method of studying the role of the gut microbiota was provided strategies to enhance the role of the gut microbiota in regulating osteoporosis and osteoarthritis. In this review, we discuss how depletion of the gut microbiota affects osteoporosis and osteoarthritis.

## Highlights

- Intestinal flora can affect joints by regulating inflammation and metabolism, but cartilage metabolism is still different from the intestinal flora.
- In this case, a variety of recombinant sciences, such as metabolomics and transcriptomics, help to decipher this complex problem through molecular resolution by combining specific metabolites, genes, or signaling pathways.
- Single-cell technology should reveal the correlation between different cell subpopulations and the gut microbiota. In addition to mechanism studies, there is evidence that inflammation and intestinal diseases may be related to new biomarkers that can predict the progression of OA and OP and monitor the effectiveness of therapeutic interventions.

## Introduction

Osteoporosis and osteoarthritis are serious diseases that threaten human health and are associated with considerable morbidity. In the past, osteoporosis and osteoarthritis were both considered skeletal diseases and not closely related to other body systems (1). Osteoporosis worldwide causes 8.9 million fractures, which means that every three seconds osteoporosis fracture occurs (2). Osteoporosis are affect 200 million women in worldwide which mean that women aged 60 was approximately one-tenth, women aged 70 was one-fifth, women aged 80 was two-fifths, and women aged 90 was two-thirds (2). A significant economic burden is associated with osteoporosis fractures (3). Osteoarthritis is also a disease that seriously endangers the health of the elderly., Osteoarthritis affect 30.8 million adults in the United States and 300 million individuals in worldwide (4). Osteoarthritis often leads to pain, dysfunction, and a decline in quality of life, which brings a serious financial burden to society. Osteoarthritis is estimated to cost $303 billion dollars annually in medical costs and lost earnings (5). Therefore, it is very important to pay attention to the prevention and treatment of osteoporosis and osteoarthritis.

Currently, there are many studies on the relationship between intestinal flora and bone metabolism (6). The interactions of the host microbiota are key factors for health and can ultimately be used to develop a stable microbial community (7). The gut microbiota can serve to provide essential vitamins; regulate the host’s intestinal epithelium, immune system, maintain the nutritional metabolism, exogenous and drug metabolism of the host, as well as the structural integrity of the intestinal mucosal barrier; pathogen resistance (8, 9). The gut microbiota has been associated with several chronic conditions, such as inflammation (10), obesity (11), and metabolic disease (12). In addition, depletion of the gut microbiota can affect both the intestines and central nervous system, indicating the existence of a microbiota-gut-brain axis (13). Obesity-associated circulating metabolites are also linked to gut microbes (14). Microbiota interacts with physiological aging processes, which can be used to develop microbiota-based health surveillance systems for older adults (15).

The gut microbiota is affected by different environments. Initially, the gut microbiota obtains radon from the mother through the birth canal (16). The gut microbiota gradually stabilizes after the age of three years old. The composition of the intestinal flora is a dynamic balance that can be affected by diet. However, the gut microbiota is subsequently shaped by medical practices and lifestyle changes (17). Many factors, such as antibiotics, C-section, washing of the skin, and oral ingestion of antibacterial agents, can change this dynamic balance (18–20). The interaction between intestinal microbes and commonly used non-antibiotic drugs is complex and mutual: the composition of the intestinal flora will be affected by the drug, and the intestinal flora will also change the structure and properties of the drug (21).

Gut microbiota can be depleted by antibiotics or can be studied in germ-free mice. Antibiotic-induced depletion of the gut microbiota also has an impact on bone metabolism (22), such as impacting bone growth and preserving the progression of osteoarthritis (23–25). Germ-free mice can also experience bone loss but reduced development of osteoarthritis (26, 27). In this essay, we review recent studies addressing the role of depletion of gut microbiota in osteoporosis and osteoarthritis and discuss the proposed mechanisms.

## The Effect of Gut Microbiota on Bone Health

There are many common mechanisms for the effects of gut microbiota on osteoporosis and osteoarthritis, and in the first part of this study, we systematically summarize the factors affecting osteoporosis and osteoarthritis, including factors that affect nutrient absorption, change hormone levels, and mediate the immune response.

### Nutrient Absorption

There were significant differences in nutrient absorption among different types of gut microbiota. For example, the probiotics L. reuteri and Bifidobacterium can promote nutrient absorption which caused the increase of BMD (28). However, this effect is usually achieved by altering the PH of the gut and thus affecting the absorption of nutrients. Intestinal microbes help break down large particles into smaller, more easily digestible particles, which are important for human bone health and metabolism (29). Vitamins B and K are also common synthesis products of the microorganisms (30, 31). The host’s diet also affects the absorption of nutrients by changing the composition of the gut microbiota. The intake of carbohydrates and other substances can provide an energy source for the gut microbiota and also alter its composition. A calorie-restricted diet is associated with increased abundance of Akkermansia spp. and Bifidobacterium spp. and depletion of the abundance of Prevotella sp which may cause bodyweight loss (32). Adding inulin to food can effectively regulate the intestinal ecosystem and increase calcium absorption (33). However, the absorption of minerals directly affects the level of minerals in circulation, which in turn has a significant impact on osteoarthritis and osteoporosis. Therefore, it is important to keep balanced between nutrient absorption and gut microbiota.

### Change Intestinal Mucosal Barrier

The intestinal mucosal barrier is vital for maintaining intestinal health which was used to resist the invasion of external pathogenic microorganisms. Besides, the composition of the intestinal flora is an important factor in regulating the barrier function of the intestinal mucosa (34). For instance, the results show that the intestinal flora is the basis for the formation of a proper layer of mucus and that the mucus of GF mice differs from that of mice that are fed conventionally (35). The expression pattern of glycosyltransferase may play an important role in influencing the intestinal flora of mucus components (36). In addition, the glycosylation of MUC2 and the glycosylation of transmembrane mucin may be affected by the host bacterial community (37). Besides, the dysfunction of the intestinal mucosal barrier may lead to an increase in serum levels of lipopolysaccharide (LPS), which could in turn increase membrane permeability, resulting in the loss of bone mass (38). Intestinal barrier and mucosal immune disorders related to intestinal dysbiosis are the main causes of systemic inflammation and progressive fibrosis (39). Therefore, the relationship between the intestinal flora and the intestinal mucosal barrier is very complicated.

### Change Hormone Levels

Host hormones not only shape the structure and function of the host microbiome, but also can change the production and regulation of host hormones (such as catecholamines, estrogen, testosterone, thyroid, and growth hormone) and change the hormone expression profile (40). In contrast, conventional antibiotic treatment in mice reduces serum IGF-1 and inhibits bone formation. Supplement of antibiotic-treated mice with microbial metabolites such as short-chain fatty acids (SCFA) can restore IGF-1 and bone mass than not treated with antibiotics (41). Intestinal microbes and their metabolites regulate bone mass through the interaction of parathyroid hormone (PTH), the immune system, and bones (42). Intestinal flora also participates in alcoholic osteoporosis in young and old rats through immune regulation (43). Intestinal flora is an important factor in the occurrence of metabolic diseases (such as obesity) and is considered to be an endocrine organ that participates in maintaining the body’s energy balance and bone metabolism (44).

### Mediate Immune Response

The composition of the intestinal flora affects the development of the immune system and regulates immune mediators, thereby affecting the intestinal barrier. Analysis of observed daily changes in the counts of neutrophils, lymphocytes, and monocytes, as well as analysis of samples from more than 10,000 longitudinal microbial communities, revealed a consistent correlation between gut bacteria and immune cell dynamics (45). T helper 17 lymphocytes, TNF, interleukin 17, and Other important pathways include NOD1, NOD2, and Toll-like receptor 5, RANK ligand which are involved in the immune response of the intestinal flora (46). Firstly, in the first few years of life, the colonization of microbial communities is essential for the optimal development of the immune system (47). Lack of microbiota, intestinal mucosal immune is undeveloped due to small mesenteric lymph nodes, decreased PP and immune cells, such as plasma cells that produce IgA, CD4+ T cells, and CD8+ T cells which leads to a weakened resistance pathogenic bacteria (48). The composition of the intestinal flora also regulates the balance between helper effector T cells, which produce the inflammatory factor interleukin 17 in the intestine (49). The microbiome metabolites affect immune regulation and autoimmunity. For instance, gut microbiota in the production of pro-inflammatory cytokines and subsequent generation of Th17 cells and also promote the generation of regulatory T cells which contribute to immune suppression (50). Due to the anaerobic fermentation of residues in the digestive system, the possibility of the microbiota to regulate host physiology is the extremely diverse metabolite pool they produce (51). The receptor activator of NFκB ligand (RANKL) is consider as the main cytokine in osteoclast differentiation which is produced by mesenchymal cells, osteoblasts, and osteocytes in the bone marrow. The activated CD4+ T cells are also a source of RANKL which was affected by gut microbiota (52, 53). The excessive bacterial proliferation in the small intestine can occur with lower bone density, possible causes related to high levels of inflammation of TNF-α and IL-1, and promote osteoclast activation (54). Therefore, understanding the regulation of the immune response of the gut microbiota may allow medical interventions on the microbiota to prevent or treat diseases related to bone metabolism.

## The Potential Mechanism of Depletion of Gut Microbiota in Osteoporosis

**Figure 1:**
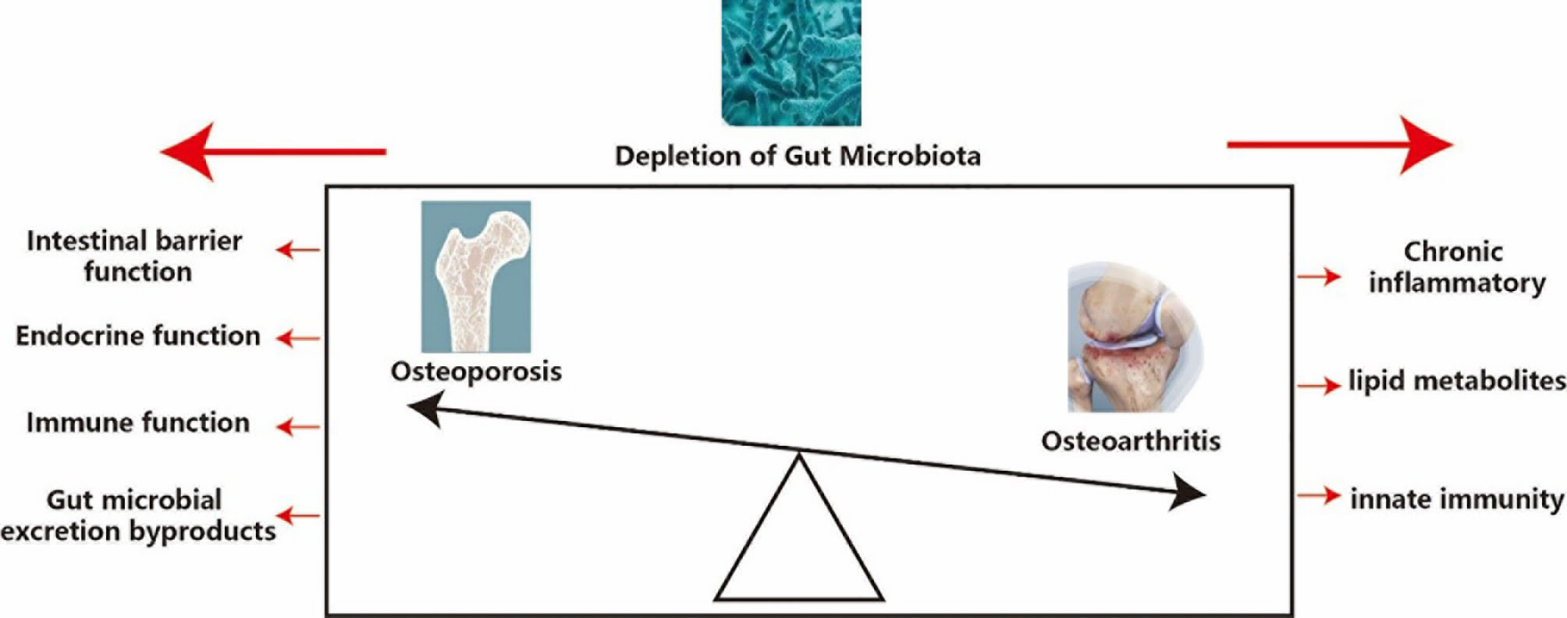
Potential mechanism of depletion of gut microbiota in osteoporosis and osteoarthritis. Osteoporosis and osteoarthritis are like the front and back sides of a coin, and depletion of gut microbiota has a significant impact on both osteoporosis and osteoarthritis, so it is important to analysis the effect of depletion of gut microbiota on osteoporosis and osteoarthritis.

There are many potential mechanisms of gut microbiota depletion in osteoporosis, such as impaired intestinal barrier function, endocrine function, immune function and gut microbial excretion byproducts. Changes in bacterial species in the gut microbiota can influence intestinal barrier function, which can affect the processes of nutrient absorption. Intestinal microbiota may affect intestinal pH (55) and affect the synthesis of vitamins B and K (56), which plays an important role in controlling calcium absorption (57). The depletion of gut microbiota in diet-induced obesity mediates changes in the ileum and allows macromolecular substances to pass quickly through the intestinal wall of the intestinal epithelium (58). However, damage to intestinal barrier function can also influence the composition of gut microbiota by increasing lipopolysaccharide (LPS) (38). Gut hormones also have many key roles in bone metabolism, which involves the accumulation of various signaling pathways, including G protein-coupled receptors, nutrient transporters, and ion channels (59). Both antibiotic-induced and germ-free models represent a basic model to understand the relationship between microbiota and the immune system by regulating T helper 17 lymphocytes, TNF, interleukin 17, and the RANK ligand system (46, 60). Intestinal microbial consumption affects host endocrine function through several bacteria-derived metabolites, including glucagon-like peptide 1 and peptide YY (61). Gut microbial excretion byproducts, for example, Glucagon-like peptide 1 is an amino acid hormone and is also secreted by endocrine L cells. It plays a regulating role in the process of osteoporosis by changing the balance between the differentiation of mesenchymal stem cells of the bone into osteocytes and adipocytes (62). Peptide YY is also a negative regulator of osteoblast bone formation (63) (**Table 1** and **Figure 1**).

**Table 1:** Gut microbiota depletion studies on osteoporosis.

Subject	Study design	Outcome	Citation
***antibiotic induced-gut microbiota depletion and osteoporosis***			
SCD mice	Compared conventionalized mice with and without antibiotic treatment	Antibiotic significantly improved decreased bone mass and impaired intestinal barrier	(65)
C57BL/6 mice	Compared conventionalized mice with and without antibiotic treatment	Increased BMD at 3 weeks but not at 7 weeks and Increased body fat but no change in weight with antibiotic treatment	(66)
C57BL/6 mice	Compared conventionalized mice with and without antibiotic treatment	Early-life antibiota treatment accelerates total mass and bone growth	(67)
C57BL/6 mice	Compared conventionalized mice with and without antibiotic treatment	Bone mineral density is increased in early-life growth with antibiota treatment	(68)
TLR5KO and WT (C57Bl/6) mice	Compared conventionalized mice with and without antibiotic treatment	antibiota treatment in the gut microbiota for extended periods during growth may lead to impaired whole-bone mechanical properties	(69)
***Gree-free induced-gut microbiota depletion and osteoporosis***			
BALB/c mice	Compared GF and conventionalized mice	germ-free (GF) mice with conventional specific pathogen-free (SPF) gut microbiota increases both bone formation and resorption	(41)
BALB/c mice	Compared GF and conventionalized mice	presence of microbiota led to Increased BMD, BVF, femur length and Increased body weight, body length	(78)
C57BL/6 mice	Compared GF and mice undergoing low-dose penicillin (LDP) treatment	Increased BMD, BMC in female mice and decreased BMC in male mice	(68)
C57BL/6 mice	Compared GF and conventionalized mice	Presence of microbiota led to decreased BMD	(27)
GF Swiss Webster and GF C57BL/6 mice	Compared GF and conventionalized mice	successful colonization of GF mice with gut microbiota of either mouse or human origin, bone mass did not change significantly in any of the groups tested.	(79)

## Evidence of Depletion of Gut Microbiota in Osteoporosis

Several studies have found that depletion of gut microbiota by antibiotic treatment affects growth in early life in mice. Antibiotics induced depletion of gut microbiota in postpubertal skeletal development by an osteoimmune response, which alters the gut bacterial composition and skeletal morphology. In addition, depletion of gut microbiota can contribute to a state of dysbiosis-mediated hyper immunity caused by inhibited the presentation of class II antigens of the major histocompatibility complex (64). Tavakoli et al. examined whether the gut microbiome contributes to bone loss in stearoyl-coenzyme desaturase mice and found that depletion of the gut microbiota significantly improved decreased bone mass. This occurred by increasing osteoblasts and osteoblast-related gene expression and impairing the intestinal barrier due to inflammation (65). Low-dose penicillin-induced depletion of gut microbiota in preadolescent mice, leading to alterations of metabolites and abnormal intestinal immunity (66). Therapeutic-dose pulsed antibiotic treatment-induced depletion of gut microbiota can accelerate body mass and bone growth (67). Cho et al. create a model of obesity in dogs by injecting subtherapeutic antibiotics and found that depletion of gut microbiota can change carbohydrate metabolism into short-chain fatty acid metabolism, providing evidence of metabolic homeostasis (68). Female bending strength was less induced by depletion of gut microbiota, and B and T cell populations were also depleted, suggesting an association between alterations in the immune cell population and bone tissue material properties (69). The effect of microbiota perturbation with antibiotics is often affected by sex, age, and the dose of antibiotic drugs (70–73).

Germ-free (GF)animals are another way to study depletion of the gut microbiota, and they can also be used to examine the effect of specific microbes on osteoporosis and osteoarthritis (74, 75). Irie et al. investigated germ-free mice to evaluate age-related immune and inflammatory systems and found that depletion of gut microbiota led to alveolar bone loss in GF mice (76). Colonization of GF mice with SPF increases bone formation, which provides IGF-1 to simulate the development of skeletal bone (41). Li et al. found that the inflammatory response is caused by a lack of steroids that regulate the intestinal microbiota, leading to loss of trabecular bone. The results show that the gut luminal microbiota increases gut permeability and triggers inflammatory pathways (77). Schwarzer et al. used Nod1−/− or Nod2−/− mice with germ-free mice to reduce bone mass (78). Quach et al. investigated the influence of different microbial communities on different mice and the success rate of fecal transplantation in sterile mice. This result shows that bone mass, bone parameters, osteoclast precursors, and T cell populations did not change significantly (79). Compared with conventional rats, GF mice increased bone mass and decreased the number of osteoclasts on the surface of each bone. This indicates that the bone immune status and the bone resorption mechanism mediated by osteoclasts have changed (27). In addition, the gut microbiota prevents excessive mineralization by enhancing osteoblast and osteoclast activity through transcription factors, such as Gata-binding protein 3 (80) (**Table 1**).

## The Potential Mechanism of Depletion of Gut Microbiota in Osteoarthritis

More and more evidence shows that some factors related to OA, such as aging, gender, diet, and obesity, can re-adjust the gut microbiota while promoting systemic inflammation. This suggests that microorganisms may participate in OA, although limited and disturbing. Convincing research confirms that this hypothesis will interfere with intestinal flora.

The possible mechanisms of depletion of gut microbiota in osteoarthritis include chronic inflammatory factors, lipid metabolites, and innate immunity. Since elevated LPS levels are associated with obesity and metabolic syndrome, and obesity and metabolic syndrome are closely related to the risk of osteoarthritis, it is easy to assume that at least one microbial community is associated with osteoarthritis, inflammation, low levels, and metabolic endotoxicity Related. Macrophage activation and joint damage (81). The increase in lipopolysaccharide (LPS) and LPS binding protein (LBP) is related to the severity of knee osteophytes and the frequency of macrophage activation in the synovium (82). In addition, an interesting study by Christopher et al. found that the characteristics of microbial DNA in human and mouse cartilage and its changes are related to the occurrence and development of human osteoarthritis (82). Because of this factor between the intestinal flora and osteoarthritis, there is an urgent need to develop effective disease-modifying therapies to relieve symptoms and slow the progression of osteoarthritis. In this case, it can be assumed that the regulation of intestinal flora by external means may affect the progression of osteoarthritis.

Fecal microbiota transplantation (FMT) is a surgical procedure used to treat diseases related to the gut microbiota, including the delivery of stool from a healthy donor to the recipient’s distal gastrointestinal tract (83). The new results show that FMT has broad application prospects in OA management. Huang et al. investigate a study to collect stool samples from healthy individuals in the OA group without metabolic syndrome and from healthy individuals in the OA group with metabolic syndrome and then transplant the collected samples to meniscus/ligament rupture osteoarthritis (MLI) Of sterile mice. Interestingly, transplantation of OA in knee joints with metabolic syndrome and MLI may lead to worsening of osteoarthritis. These results indicate that the weak microbiome in the mouse model may exacerbate the histopathological severity of osteoarthritis due to joint damage. This study illustrates the application of FMT in the study of the pathogenesis of osteoarthritis, and also provides hope for manipulating the intestinal flora to treat osteoarthritis. However, due to the limited sample size, more convincing research is needed (84).

## Evidence of Depletion of Gut Microbiota in Osteoarthritis

Depletion of gut microbiota OA caused by antibiotics has also been investigated, and this depletion can alleviate the progression of osteoarthritis (25). Depletion of the gut microbiota can also slow osteoarthritis outcomes by reducing the state of inflammation and lowering the expression of Wnt signaling modulatory proteins (85). In addition, due to the instability of the medial meniscus, depletion of the intestinal microflora in GF mice may reduce the incidence of osteoarthritis (26), which can regulate inflammation associated with the innate immune system (86).

A previous study found that obesity-associated OA can also be affected by the depletion of gut microbiota (87). In addition, lipopolysaccharide also plays an important role in the pro-inflammatory response of the lack of intestinal flora caused by gram-negative bacteria (88). Ulici et al. found that depletion of gut microbiota induced by germ-free mice could also reduce the development of osteoarthritis, which may have been caused by the lower level of lipopolysaccharide (26). Lipid metabolites also play an important role in destroying intestinal flora (44). Serum and synovial fluid lipodystrophy are also important predictors in the development of osteoarthritis (89) (**Table 2**).

**Table 2:** Gut microbiota depletion studies on osteoarthritis.

Subject	Study design	Outcome	Citation
C57BL/6 mice	Compared conventionalized mice with and without antibiotic treatment	Antibiotic-induced intestinal microbiota depletion improvement of OA after joint injury	(25)
C57BL/6 mice	Compared conventionalized mice with and without antibiotic treatment	Antibiotic Treatment Prior to Injury Improves Post-Traumatic Osteoarthritis Outcomes in Mice	(85)
C57BL/6 mice	Compared GF and conventionalized mice	Gree free status was protective against early OA changes in bone structure.	(86)
C57BL/6 mice	Compared GF and conventionalized mice	Only the microbiota transplantation from the knee OA with metabolic syndrome and MLI resulted in an increase in the severity of OA	(84)

Although depletion of gut microbiota is increasingly valued in the study of osteoarthritis and osteoporosis, it is not yet available as a means of treatment. many studies found that potential benefits of probiotic supplementation such as L. paracasei, L. casei 393-fermented milk, L. helveticus-fermented milk, L. casei and L. acidophilus and Enterococcus faecium on bone health in both healthy and pathological states which regulate of luminal pH; secretion of antimicrobial peptides; enhancement of barrier function by increasing mucus production and modulation of the host immune system (90).

## Conclusion

Osteoporosis and osteoarthritis are major public health problems and the main cause of global pain, disability, fracture risk, and socio-economic costs. Therefore, determining the pathogenesis of osteoporosis and osteoarthritis is essential for the development of new therapeutic interventions to prevent and alleviate the disease.

As an important way to regulate bone metabolism, the gut microbiota has attracted increasing attention. Depletion of the gut microbiota as a method to study its function still lacks in-depth research. In this review, we summarized the evidence supporting the gut-joint axis hypothesis and the interaction of gut microbiota with osteoarthritis or surgical factors and suggested the prospects for use of gut microbiota in the treatment of osteoarthritis and surgery Therefore, it is important to further analyze the role of gut microbiota depletion in osteoporosis and osteoporosis.

The detailed mechanism of the gut-joint axis is unclear. Intestinal flora can affect joints by regulating inflammation and metabolism, but cartilage metabolism is still different from the intestinal flora. In this case, a variety of recombinant sciences, such as metabolomics and transcriptomics, help to decipher this complex problem through molecular resolution by combining specific metabolites, genes, or signaling pathways. In addition, single-cell technology should reveal the correlation between different cell subpopulations and the gut microbiota. In addition to mechanism studies, there is evidence that inflammation and intestinal diseases may be related to new biomarkers that can predict the progression of OA and OP and monitor the effectiveness of therapeutic interventions.

## Author Contributions

Conception and design: ZYG and KT. Acquisition, analysis, and interpretation of the data: ZYG, SFL, XL, LYL, QGZ and ZQG. Drafting and writing: KT. All authors contributed to the article and approved the submitted version.

## Funding

This work was partly supported by the Clinical research project of Shanghai Tenth People’s Hospital (Grants no. YNCR2C027).

## Conflict of Interest

The authors declare that the research was conducted in the absence of any commercial or financial relationships that could be construed as a potential conflict of interest.

## Publisher’s Note

All claims expressed in this article are solely those of the authors and do not necessarily represent those of their affiliated organizations, or those of the publisher, the editors and the reviewers. Any product that may be evaluated in this article, or claim that may be made by its manufacturer, is not guaranteed or endorsed by the publisher.
